# A pilot controlled trial of a combination of dense cranial electroacupuncture stimulation and body acupuncture for post-stroke depression

**DOI:** 10.1186/1472-6882-14-255

**Published:** 2014-07-19

**Authors:** Sui-Cheung Man, Ben H B Hung, Roger M K Ng, Xiao-Chun Yu, Hobby Cheung, Mandy P M Fung, Leonard S W Li, Kwok-Pui Leung, Kei-Pui Leung, Kevin W Y Tsang, Eric Ziea, Vivian T Wong, Zhang-Jin Zhang

**Affiliations:** 1 The School of Chinese Medicine, The University of Hong Kong, 10 Sassoon Road, Pokfulam, Hong Kong, China; 2 Department of Psychiatry, Kowloon Hospital, Hong Kong, China; 3 Institute of Acupuncture, China Academy of Chinese Medical Sciences, Dongchen District, Beijing 100700, China; 4 Department of Rehabilitation, Kowloon Hospital, Hong Kong, China; 5 Rehabilitation Unit, Tung Wah Hospital, Hong Kong, China; 6 Department of Medicine and Rehabilitation, Tung Wah Eastern Hospital, Hong Kong, China; 7 Chinese Medicine Department, Hospital Authority, Hong Kong, China

**Keywords:** Acupuncture, Post-stroke depression, Rehabilitation, Dense cranial electroacupuncture stimulation, DCEAS, Clinical trial

## Abstract

**Background:**

Our previous studies have demonstrated the treatment benefits of dense cranial electroacupuncture stimulation (DCEAS), a novel brain stimulation therapy in patients with major depression, postpartum depression and obsessive-compulsive disorder. The purpose of the present study was to further evaluate the effectiveness of DCEAS combined with body acupuncture and selective serotonin reuptake inhibitors (SSRIs) in patients with post-stroke depression (PSD).

**Methods:**

In a single-blind, randomized controlled trial, 43 patients with PSD were randomly assigned to 12 sessions of DCEAS plus SSRI plus body electroacupuncture (n = 23), or sham (non-invasive cranial electroacupuncture, n-CEA) plus SSRI plus body electroacupuncture (n = 20) for 3 sessions per week over 4 weeks. Treatment outcomes were measured using the 17-item Hamilton Depression Rating Scale (HAMD-17), the Clinical Global Impression - Severity scale (CGI-S) and Barthel Index (BI), a measure used to evaluate movement ability associated with daily self-caring activity.

**Results:**

DCEAS produced a significantly greater reduction of both HAMD-17 and CGI-S as early as week 1 and CGI-S at endpoint compared to n-CEA, but subjects of n-CEA group exhibited a significantly greater improvement on BI at week 4 than DCEAS. Incidence of adverse events was not different in the two groups.

**Conclusions:**

These results indicate that DCEAS could be effective in reducing stroke patients’ depressive symptoms. Superficial electrical stimulation in n-CEA group may be beneficial in improving movement disability of stroke patients. A combination of DCEAS and body acupuncture can be considered a treatment option for neuropsychiatric sequelae of stroke.

**Trial registration:**

http://www.clinicaltrials.gov, NCT01174394.

## Background

Mood depression is a common and serious consequence of stroke. About 30% of stroke patients would develop post-stroke depression (PSD), either in the early or late stage after stroke onset [1]. Although PSD is strongly associated with poor prognosis and increased disability, it is often overlooked by clinicians. Only a small portion of patients are properly diagnosed and receive treatment [2,3]. Antidepressants, typically represented by selective serotonin reuptake inhibitors (SSRIs) and heterocyclics, are the mainstays of treatment for PSD [4,5]. Several controlled studies have demonstrated that while some patients significantly improved on depression scores, others discontinued due to side effects [4,5]. These side effects may also aggravate patients’ morbidity [1]. On the other hand, the use of antidepressants might also increase the risk of drug-drug interactions, as most stroke patients are concurrently taking drugs of other classes [6].

Apart from the general understanding that acupuncture is effective in chronic pain treatment [7], its efficacy for post-stroke conditions have also been investigated. A meta-analysis reported the therapeutic benefits of acupuncture on stroke related disability [8]. Another study reported acupuncture as safe and effective in treating PSD [9]. In a recent systematic review and meta-analysis it has been suggested that acupuncture might be superior to antidepressants and waitlist controls in improving clinical response and reducing depressive symptoms of PSD patients [9]. However, as a number of these trials lacked methodological vigour, further appropriately design clinical studies are recommended to verify the conclusions.

Recently we have developed a novel brain stimulation called dense cranial electroacupuncture stimulation (DCEAS), in which electrical stimulation is directly delivered on a group of acupoints on the forehead, which are innervated by the trigeminal sensory pathway [10]. The benefits of DCEAS have been identified in the treatment of major depression [11], postpartum depression [12] and obsessive-compulsive disorder (OCD) [13]. In a recent clinical trial, it has been demonstrated that electrical stimulation on scalp acupoints would produce a long-lasting enhancement of antidepressant efficacy [14]. These were the reasons to hypothesize that electroacupuncture could improve clinical outcomes in patients with PSD.

The objective of the present study was to determine whether DCEAS combined with SSRIs and body acupuncture could produce better clinical outcomes in PSD subjects, as compared to sham (non-invasive cranial electroacupuncture, n-CEA). To test this hypothesis, this single-blind, randomized, sham-controlled clinical study was designed.

## Methods

### Settings and subjects

This single-blind, randomized, sham-controlled trial was conducted in Division of Rehabilitation Medicine of Tung Wah Hospital, Division of Rehabilitation Medicine of Tung Wah Eastern Hospital, Department of Psychiatry and Department of Rehabilitation of Kowloon Hospital at Hong Kong between February 2011 and August 2012. The study protocol was approved by Institutional Review Board (IRB) of the University of Hong Kong/Hospital Authority Hong Kong West Cluster and registered in http://www.clinicaltrials.gov (NCT01174394). The reporting of the trial would adhere to the CONSORT 2010 statement [15]. The CONSORT checklist and flowchart were included as Additional files 1 and 2.

Patients who met all of the following criteria were eligible for the study: (1) men or women aged 35 to 80 years old; (2) diagnosed as ischemic or haemorrhagic stroke within 6 months, confirmed with cerebral computed topographic scanning or magnetic resonance imaging; and (3) developed significant depressive episode, with score of 16 or greater in the 17-item Hamilton Rating Scale for Depression (HAMD-17) [16].

Patients who met any of the following criteria were excluded from the study: (1) presence of severe aphasia, especially fluent aphasia; (2) presence of severe cognitive dysfunction, as indicated by the Mini-mental State Examination (MMSE) score < 18 [17]; (3) history of psychiatric illness other than depression; (4) presence of another chronic disorder, including severe Parkinson’s disease, cardiac disease, cancers, epilepsy, or chronic alcoholism; (5) having impaired hepatic or renal function; or (6) having bleeding tendency.

All participants were able to give voluntary, written and informed consent on their own before entering the trial.

### Randomization and blinding

Subjects of both groups would receive SSRIs and body electroacupuncture as conventional treatment. They were randomly assigned to receive DCEAS or n-CEA (sham). For randomization, simple, complete, non-sequential random numbers were generated in advance by a computer program (SPSS version II) in a block of four, and kept by the Principal Investigator (PI, ZJZ). After a patient’s eligibility was confirmed, a randomization number which corresponded to the group allocation would be provided to the acupuncturist (BHBH) by PI. Such arrangement would ensure the clinical assessor (MSCM) and subjects to be blind to the allocation.

### Treatment procedures

#### SSRI treatment regimens

Subjects of both groups received orally administered SSRIs for 4 weeks in an open manner. For those who were currently under SSRIs treatment, they would continue their existing treatment regimens. For those who were not medicated at the time of trial, fluoxetine (FLX) was given at an initiate dose of 10 mg/day and escalated to an optimal dose within one week, based on individual patient response, and the maximum dose was set at 40 mg/day. In the week of FLX initiation, acupuncture procedure also started spontaneously (see below). FLX was selected because it is one of the most frequently prescribed SSRIs worldwide [18], and it can enhance motor recovery in ischaemic stroke patients [19]. Subjects who had poor compliance with medication schedules (below 80%) would be removed from the study. Concomitant use of other psychoactive agents was generally not allowed, but the use of benzodiazepines for insomnia was permitted as long as these were taken no more than 14 days cumulatively.

#### Acupuncture intervention

Acupuncture intervention was conducted for 3 sessions per week over 4 consecutive weeks while subjects were being medicated with SSRIs. The 4-week treatment duration was designated because robust effects of acupuncture in stroke patients were generally observed within this period [9]. Both groups of subjects received body electroacupuncture by electrically stimulating between ipsilateral Hegu (LI4) and Quchi (LI11) and between ipsilateral Zusanli (ST36) and Taichong (LR3) on both sides. These body acupoints were commonly used in the acupuncture for stoke treatment. Meanwhile, subjects assigned to DCEAS received electrical stimulation on the following 6 pairs of cranial acupoints: Baihui (GV20) and Yintang (EX-HN3), left Sishencong (EX-HN1) and Toulinqi (GB15), right Sishencong (EX-HN1) and Toulinqi (GB15), bilateral Shuaigu (GB8), bilateral Taiyang (EX-HN5), and bilateral Touwei (ST8). For electrical stimulation on limbic and cranial acupoints, disposable acupuncture needles (0.30 mm in diameter and 25–40 mm in length) were inserted at a depth of 10–30 mm obliquely into limbic and cranial acupoints, on which electrical stimulation with continuous waves with 2 Hz at 9 volts were delivered through an electrical acupuncture stimulation instrument (Hwarto, SMY-10A). The use of the low frequency rather high frequency was adopted because it could induce biochemical changes in the brain in more favourable manner for alleviating depressive symptoms [20,21]. The intensities of stimulation were adjusted to a level at which subjects felt most comfortable. The stimulation lasted 30 min.

For subjects assigned to n-CEA, Streitberger’s non-invasive acupuncture needles were applied to serve as sham control at the same cranial acupoints with the same stimulation modality, except that the needles were only adhered to the skin instead of insertion [22]. Its validity and credibility has been well demonstrated [22-24]. Briefly, needles without sharp tips were quickly put onto the acupoints as used in DCEAS. The needles did not actually pierce through the skin, though subjects would feel like being treated. The needles were then affixed on the skin with adhesive tapes. Since all the cranial acupoints were beyond subjects’ vision, they could not visualize the acupuncture procedure. This sham acupuncture modality has also been adopted in our previous studies [11-13], without any problems encountered.

#### Rehabilitation therapy

In view of the fact that a large proportion of stroke patients had physical rehabilitation treatment, subjects participating in the study would be allowed to continue their designated physical rehabilitation therapy. Whether subjects had had rehabilitation therapy would be included in statistical analysis as a covariate (see below).

### Clinical assessment

Subjects’ cognitive function was measured using MMSE during screening. Those with MMSE score less than 18 were excluded from the study as mentioned in the inclusion criteria. The primary efficacy was measured using the HAMD-17, CGI-S, and Barthel Index (BI). BI is a concise index of independence to score movement ability of a patient in caring himself/herself [25]. Adverse events were assessed using the Treatment Emergent Symptom Scale (TESS) [26]. Assessments were conducted at baseline, week 1, 2 and 4. To ensure consistency and reliability of assessments across the study, assessors received a training session. A reliability coefficient of ≥ 0.80 was required to be achieved after the training session on HAMD-17 and BI. Assessments for a patient were generally completed by the same assessor.

To check the credibility of the n-CEA and DCEAS procedures, based on the method by Flink et al. [24], each subject was asked the same question at the end of the last clinical assessment: “As we informed you that you had an equal chance of receiving sham or active acupuncture treatment, which do you think you had received?”.

### Data analysis

Previous studies have shown that acupuncture intervention could produce at least a 4-point reduction in HAMD-17 in PSD patients [9]. Therefore, a sample size of 40 subjects (n = 20 per group) should provide an approximately 80% power at a statistical level of 0.05, with an estimated standard deviation of 4.0 and an estimated dropout rate of 15% at the endpoint of treatment.

Efficacy analyses were performed on the intention-to-treat population, defined as participants who completed baseline and at least one evaluation after treatment. A linear mixed-effect model was applied to compare treatment outcomes (HAMD-17, CGI, and BI) over time between the two groups. The model was established using time and group for categorical fixed factors and random intercepts with scaled identity covariance matrix. Subject’s age, gender, duration of the illness, baseline HAMD-17 and BI were treated as covariates. Between-group differences at each measure time point were further examined using Student *t* test. Student *t* test was also applied to detect between-group differences in continuous baseline variables. Categorical variables, including categorical baseline variables and incidence of adverse events were analyzed using Chi-square (*χ*^2^) or Fisher Exact test. Statistical significance was defined as a two-tailed *P*< 0.05. The analyses were performed with SPSS version 16 software (Chicago, IL, USA).

## Results

### Baseline characteristics of subjects

**Figure 1:**
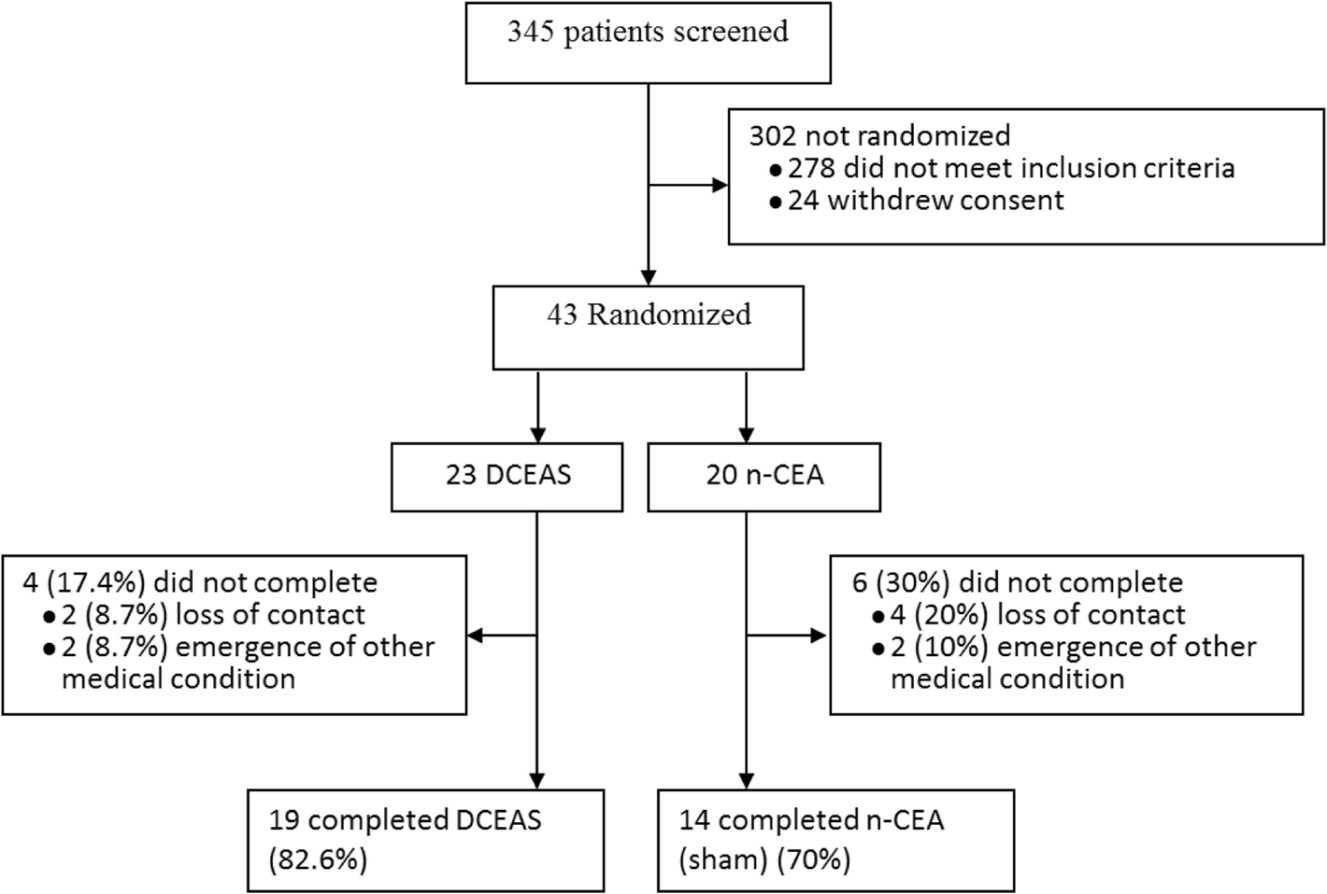
**Enrollment flow.** Of 345 patients screened, 43 eligible subjects were randomly assigned to n-CEA (sham) (n = 20) and DCEAS (n = 23) group. For n-CEA (sham), 14 subjects completed; while for DCEAS, 19 completed.

Of 345 patients screened, 43 eligible subjects were randomly assigned to n-CEA (sham) (n = 20) and DCEAS (n = 23) group and 33 (77%) completed the 4-week treatment, but all subjects completed baseline and at least one evaluation after treatment. All 43 subjects were therefore included in data analysis (Figure 1). There were no significant differences between the two groups in baseline variables. All subjects had received physical rehabilitation therapy when they entered the study. A majority of subjects (86%) were having SSRIs treatment at study entry and 78% of them were treated with FLX. All subjects had received rehabilitation therapy with 1–2 sessions when they entered the study. Cognitive function measured with MMSE was similar in the two groups (Table 1). The compliance with acupuncture and SSRI treatment was approximately nearly 95% in the two groups.

**Table 1:** Baseline characteristics of subjects

**Variables**	**n-CEA (n = 20)**	**DCEAS (n = 23)**	***P *** **value**^**b**^
Female, n (%)	13 (65.0)	14 (60.9)	0.780
Age (y)^a^	66.5 ± 11.9	66.7 ± 13.6	0.969
Having previous stroke, n (%)	2 (10.0)	6 (26.1)	0.250
Post-stroke duration (m)^a^	10.2 ± 14.9	7.7 ± 11.6	0.547
Duration of PSD onset (m)^a^	4.9 ± 12.0	4.7 ± 10.7	0.953
Having antidepressant treatment at study entry, n (%)^c^			0.222
Fluoxetine (FLX)	14 (70.0)	15 (65.2)	
Other antidepressant agents^d^	5 (25.0)	3 (13.0)	
Previous acupuncture experience, n (%)	14 (70.0)	13 (56.5)	0.362
Baseline MMSE^a^	23.5 ± 4.4	24.8 ± 4.8	0.354
Baseline HAMD-17^a^	28.1 ± 7.0	25.8 ± 5.0	0.225
Baseline CGI-S^a^	4.8 ± 0.8	4.9 ± 0.5	0.580
Baseline BI^a^	37.8 ± 10.7	37.7 ± 11.3	0.975

^a^Continuous data are expressed as mean ± SD.

^b^Student *t* test and Fisher Exact test were used to detect between-group differences of continuous and categorical data, respectively.

^c^Duration of antidepressant treatment largely varied from 10 days to 2 years.

^d^Other antidepressants were sertraline, citalopram, escitalopram, mirtazapine and flupentixol + melitracen.

*BI* Barthel Index, *CGI-S* Clinical Global Impression-Severity, *DCEAS* Dense Cranial Electroacupuncture Stimulation, *HAMD-17* 17-item Hamilton Rating Scale for Depression, *MMSE* Mini-Mental Status Examination, *n-CEA* Non-invasive Cranial Electroacupuncture, *PSD* Post-stroke Depression.

### Credibility of n-CEA and DCEAS

For subjects treated with n-CEA, 90% (18/20) of them perceived to have received DCEAS, while 13.0% (3/23) subjects in DCEAS group perceived to have received n-CEA treatment. Using fisher exact test, it yielded no significant difference in the credibility rating (p = 1.0).

### Efficacy

**Figure 2:**
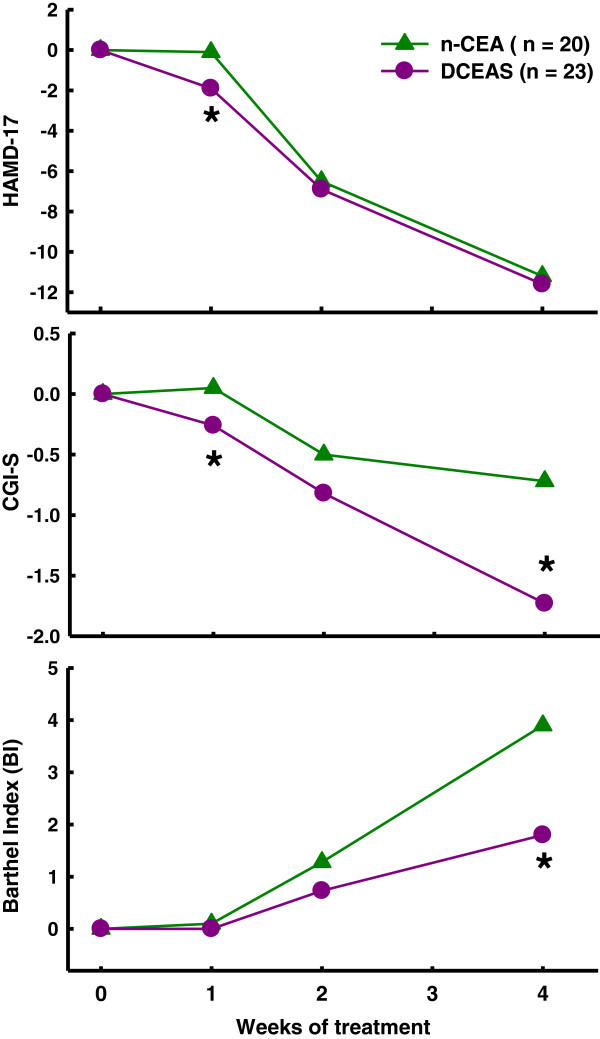
Changes in score from baseline on HAMD-17, CGI-S, and BI over time.

Changes in score from baseline on HAMD-17, CGI-S, and BI over time are illustrated in Table 2 and Figure 2. Linear mixed-effects model revealed a significant difference on the intercept between n-CEA and DCEAS groups in CGI-S (*F* = 14.220, df = 1,169, *P* = 0.0002), and on the slope in BI (*F* = 24.101, df = 1,168, *P*< 0.0001). There was a borderline significance on the slope in CGI-S (*F* = 3.716, df = 1,168, *P* = 0.0556). No significant differences in HAMD-17 were observed between the two groups on either slope (*F* = 0.031, df = 1,168, *P* = 0.86) or intercept (*F* = 3.224, df = 1,169, *P* = 0.0744). Between-group comparisons exhibited that DCEAS-treated subjects had a significantly greater reduction in HAMD-17 at week 1 (*P* = 0.007) and CGI-S at week 1 (*P* = 0.001) and week 4 (*P* = 0.001). Significantly greater improvement on BI was observed in subjects receiving n-CEA at week 4 (*P*< 0.001) compared to DCEAS group.

**Table 2:** Changes in score on depression scales from baseline in PSD subjects

**Variables**	**n-CEA**	**DCEAS**	**Slope**		**Intercept**	
	**(n = 20)**	**(n = 23)**	***F***	***P***	***F***	***P***
HAMD-17			0.031	0.860	3.223	0.074
Week 1	-0.1 ± 1.9	-1.9 ± 2.3**				
Week 2	-6.5 ± 1.9	-6.8 ± 2.3				
Week 4	-11.2 ± 1.9	-11.6 ± 2.3				
CGI-S			3.716	0.056	14.220	<0.001
Week 1	0.1 ± 0.3	-0.3 ± 0.3**				
Week 2	-0.7 ± 0.3	-0.8 ± 0.3				
Week 4	-1.4 ± 0.3	-1.7 ± 0.3**				
BI			24.101	<0.001		
Week 1	0.1 ± 1.2	0.0 ± 0.8				
Week 2	1.3 ± 1.2	0.7 ± 0.8				
Week 4	3.9 ± 1.2	1.8 ± 0.8**				

^a^Overall and between-group *P* values were obtained from linear mixed-effects model analysis and student *t*-test, respectively. **P< 0.01 compared to n-CEA group.

*BI* Barthel Index, *CGI-S* Clinical Global Impression-Severity, *DCEAS* Dense Cranial Electroacupuncture Stimulation, *HAMD-17* 17-item Hamilton Rating Scale for Depression, *n-CEA* Non-invasive Cranial Electroacupuncture, *PSD* Post-stroke Depression.

### Medication and rehabilitation profile

There was no significant difference in the average dose of FLX taken during 4 weeks of treatment between DCEAS-treated and n-CEA groups (21.2 ± 8.0 mg/day vs. 21.8 ± 8.1 mg/day, mean ± SD, *P* = 0.820). The proportion of subjects medicated with benzodiazepines for insomnia was not significantly different, with 39% (9/23) for DCEAS group and 55% (11/20) for n-CEA group (*P* = 0.366). All subjects continued their rehabilitation with 1–2 sessions per week.

### Safety and tolerability

A total of 12 reported adverse events occurred in at least 5% of subjects in either group are summarized in Table 3. No significant differences were found between the two groups in the incidence of any adverse events. None of the subjects discontinued their treatments due to adverse events.

**Table 3:** **Adverse events occurred in at least 5% of subjects in either group**^**a**^

**Event**	**n-CEA (n = 20)**	**DCEAS (n = 23)**
Fatigue	8 (40.0)	11 (47.8)
Needling-induced discomfort	6 (30.0)	7 (30.4)
Headache	6 (30.0)	4 (17.4)
Dizziness	1 (5.0)	4 (17.4)
Nausea	2 (10.0)	1 (4.3)
Vomiting	0	2 (8.7)
Depression deterioration	2 (10.0)	3 (13.0)
Movement disorder	3 (15.0)	5 (21.7)
Sweating	1 (5.0)	1 (4.3)
Palpitation	1 (5.0)	5 (21.7)
Insomnia	3 (15.0)	6 (26.1)
Hypersomnia	2 (10.0)	8 (34.8)

^a^There were no significant differences in any adverse event between the two groups, as examined with Fisher Exact test.

*DCEAS* dense cranial electroacupuncture stimulation, *n-CEA* non-invasive cranial electroacupuncture.

## Discussions

The present study showed that DCEAS treatment produced a significantly greater reduction on HAMD-17 at week 1 and CGI-S at week 1 and week 4 compared to n-CEA controls, although the effects were small. The incidence of adverse events was not different in the two groups; there were no subjects who discontinued treatments due to intolerance to needling stimulation. These results are highly consistent with our recent trials of DCEAS in major depression [11] and postpartum depression [12], indicating that DCEAS is effective in rapidly reducing PSD of stroke patients. Our most recent study has further showed that electrical stimulation on scalp acupoints even produced a long-lasting enhancement of antidepressant effects in patients with major depression [14]. However, we noticed that the magnitude of DCEAS effects in reducing PSD observed in the present study was smaller than that that in our previous trials. This may be largely due to relatively smaller sample size.

It was interesting to observe that subjects of n-CEA group had better treatment outcomes than DCEAS group on movement disability associated with daily self-caring activity. As for n-CEA, though the needle did not pierce through the skin, similar electrical stimulation was also applied. The superficial electrical stimulation on the scalp appeared to be more beneficial in improving limbic paralysis. It is known that many forms of non-invasive brain stimulation, including transcutaneous electrical nerve stimulation (TENS), mainly excite mechanoreceptors and thick myelinated afferent nerve fibers, such as Aβ and Aδ; whereas DCEAS is a noxious stimulation that mainly excites noxious receptors of the scalp [10]. The activation of mechanoreceptors and thick myelinated afferent nerve fibers appeared to be more efficacious in alleviating locomotor impairment [10]. This could probably explain the clinical empiricism that physiotherapy and traditional Chinese medicine (TCM) massage (tui-na) are generally more effective in rehabilitating limbic paralysis of stroke patients. The paradoxical effects of DCEAS in improving depression and post-stroke disability also connoted that the greater improvement of DCEAS on PSD was unlikely due to the improvement of movement ability. This is in line with a higher prevalence of depressive symptoms of stroke patients than orthopaedic patients with similar level of movement disability [17].

There should be a specific neural mechanism responsible for the effects of DCEAS in reducing PSD. Stroke not only results in the primary ischemic lesion, but also interrupts central neurotransmitter functions [27], in particular the brainstem serotonin (5-HT) and noradrenaline (NA) neuronal systems, which play a pivotal role in modulating mood activity and processing acupuncture signals [10]. DCEAS was developed mainly based on a neurobiological rationale. It is well documented that the forehead acupoints innervated by the trigeminal sensory pathway have intimate connections with the brainstem reticular formation, in particular the dorsal raphe nucleus (DRN) and the locus coeruleus (LC) [10]. The latter two brain structures are the major resources of 5-HT and NA neuronal bodies, respectively, sending diffuse projections to subcortical and cortical areas, including the prefrontal cortex and the amygdala known to be heavily involved in the pathogenesis of depression [10]. Neuroimaging studies have shown that electroacupuncture stimulation is capable of directly modulating the activity of the emotion processing-related brain regions [28]. Through the intimate collateral connection from the trigeminal sensory pathway to the brainstem 5-HT and NA neuronal systems, the needling of the forehead acupoints with subsequent electrical stimulation could more efficiently elicit afferent acupuncture signals via biophysical and biochemical reactions at local acupoints and, in turn, modulates central 5-HT and NA neuronal functions [10]. Our recent study further showed that active acupuncture results in lateralisation of functional cerebral response to the contralateral unaffected hemisphere in patients with unilateral stroke, suggesting that acupuncture could enhance a compensatory process by redistributing functions to the unaffected hemisphere [29].

There are several limitations in the present study. First, electrical stimulation was directly delivered on the scalp in both n-CEA and DCEAS procedures. This may elicit a transcutaneous and/or transcranial effect; however, such effect should be minimal as the stimulation intensity used in both n-CEA and DCEAS was much lower than other brain stimulations, such as repetitive transcranial magnetic stimulation (rTMS), electroconvulsive therapy (ECT) and TENS [30,31]. Therefore, the greater effects of DCEAS are most likely to be derived from biophysical and biochemical actions of needling with subsequent electrical stimulation [10]. Second, similar to most previous studies [9], the determination of body acupoints used in the present study was basically based on empirical evidence. Empirical treatment regimens have resulted in a large variation in acupuncture protocols and difficulties in comparing treatment outcomes among trials. Finally, although the present study demonstrated the antidepressant efficacy of acupuncture therapy, the underlying mechanisms are not yet well delineated. Previous studies have suggested that the antidepressant effects of acupuncture may be associated with the restoration of decreased neuroimaging activity of brain regions involved in processing emotional signals [10]. The determination of neuroimaging correlates of the clinical improvement in depressed patients would help gain some insights into antidepressant mechanisms of acupuncture.

## Conclusions

These results indicate that DCEAS could be effective in reducing stroke patients’ depressive symptoms. Superficial electrical stimulation in n-CEA group may be beneficial in improving movement disability of stroke patients. A combination of DCEAS and body acupuncture can be considered a treatment option for neuropsychiatric sequelae of stroke.

## Abbreviations

5-HT: Serotonin; BI: Barthel Index; CGI-S: Clinical Global Impression - Severity scale; CONSORT: Consolidated Standards of Reporting Trials; DCEAS: Dense cranial electroacupuncture stimulation; DRN: Dorsal raphe nucleus; ECT: Electroconvulsive therapy; FLX: Fluoxetine; HAMD-17: 17-item Hamilton Depression Rating Scale; IRB: Institutional Review Board; LC: Locus coeruleus; MMSE: Mini-mental State Examination; NA: Noradrenaline; n-CEA: Non-invasive cranial electroacupuncture; OCD: Obsessive-compulsive disorder; PI: Principal Investigator; PSD: Post-stroke depression; rTMS: Repetitive transcranial magnetic stimulation; SSRIs: Selective serotonin reuptake inhibitors; TCM: Traditional Chinese medicine; TENS: Transcutaneous electrical nerve stimulation.

## Competing interests

The authors declare that they have no competing interests in the study.

## Authors’ contributions

Conceived and designed the experiments: ZJZ, HC, RN, LSWL, KPL, KPL, VTW, EZ. Performed the experiments: SCM, BHBH, MPMF, HC, RN, KPL, KWYT. Analyzed the data: BHBH, SCM, ZJZ, XCY. Wrote the paper: BHBH, SCM, ZJZ. All authors read and approved the final manuscript.

## Pre-publication history

The pre-publication history for this paper can be accessed here:

http://www.biomedcentral.com/1472-6882/14/255/prepub

## Supplementary Material

Additional file 1 CONSORT 2010 checklist of information to include when reporting a randomised trial. Click here for file

Additional file 2 CONSORT 2010 Flow Diagram. Click here for file
