# Supplementary material for: A pilot controlled trial of a combination of dense cranial electroacupuncture stimulation and body acupuncture for post-stroke depression
Source: BMC Complement Altern Med. 2014 Jul 19;14:255. doi: 10.1186/1472-6882-14-255 (PMC4223407; doi:10.1186/1472-6882-14-255)
Supplement: Additional file 2 — CONSORT 2010 Flow Diagram. [file 1472-6882-14-255-S2.doc]

**CONSORT 2010 Flow Diagram**

**Allocation**

**Analysis**

**Follow-Up**

**Enrollment**

Assessed for eligibility (n=345)

Excluded (n=302)

♦  Not meeting inclusion criteria (n=278)

♦  Declined to participate (n=24)

♦  Other reasons (n=0)

Analysed (n=23)
♦ Excluded from analysis (n=0)

Lost to follow-up (loss of contact) (n= 2 )

Discontinued intervention (emergence of other medical conditions) (n=2)

Allocated to DCEAS (n= 23)

♦ Received allocated intervention (n=19)

♦ Did not receive allocated intervention (give reasons) (n=4)

Lost to follow-up (loss of contact) (n=4)

Discontinued intervention (emergence of other medical conditions) (n=2)

Allocated to n-CEA (n= 20)

♦ Received allocated intervention (n=14)

♦ Did not receive allocated intervention (give reasons) (n=6)

Analysed (n=20)
♦ Excluded from analysis (n=0)

Randomized (n=43)
